# Differential Expression and Target Gene Analysis of PBMC-Derived microRNAs as Prognostic Biomarkers in Acute Lymphoblastic Leukemia

**DOI:** 10.3390/ijms27093868

**Published:** 2026-04-27

**Authors:** Fatemah S. Basingab, Hadil Alahdal, Deemah Alwadaani, Ghaida Almuneef, Ahmed S. Barefah, Ali H. Algiraigri, Rawan Hammad, Mohamed Elnakeeb, Jehan S. Alrahimi, Kawther A. Zaher, Alia M. Aldahlawi

**Affiliations:** 1 Department of Biological Sciences, Faculty of Science, King Abdulaziz University, Jeddah 21585, Saudi Arabia; 2 Immunology Unit, King Fahd Medical Research Center, King Abdulaziz University, Jeddah 21859, Saudi Arabia; 3 Department of Biology, Faculty of Science, Princess Nourah University, Riyadh 11671, Saudi Arabia; 4 Medical Genomics Research Department, King Abdullah International Medical Research Center (KAIMRC), Riyadh 11481, Saudi Arabia; 5 Artificial Intelligence and Data Management Department, King Abdullah International Medical Research Center, Riyadh 11481, Saudi Arabia; 6 Hematology Department, Faculty of Medicine, King Abdulaziz University Hospital, King Abdulaziz University, Jeddah 21589, Saudi Arabia; 7 Hematology Research Unit, King Fahd Medical Research Center, King Abdulaziz University, Jeddah 21585, Saudi Arabia; 8 Department of Medical Laboratory Sciences, Faculty of Applied Medical Sciences, King Abdulaziz University, Jeddah 21589, Saudi Arabia

**Keywords:** acute lymphoblastic leukemia, microRNA, differential expression, validated targets, multiMiR, regulatory network, KEGG enrichment

## Abstract

Acute lymphoblastic leukemia (ALL) is a clinically diverse cancer in which microRNA (miRNA)-mediated post-transcriptional regulation contributes to leukemogenesis and subtype heterogeneity. In this study, miRNA expression profiling by microarray was performed on ALL cases (B-ALL and T-ALL) and healthy controls. Data were normalized and analyzed for differential expression using false discovery rate (FDR)-adjusted *p*-values. Differentially expressed miRNAs were further examined using unsupervised visualization to assess overall disease-related expression patterns. To explore their biological significance, experimentally validated miRNA–target interactions were obtained using multiMiR, limited to validated databases (miRTarBase, TarBase, and miRecords) and summarized via target-burden ranking, miRNA–target network analysis, and Circos–style interaction mapping. A unique miRNA expression signature was identified in ALL. Upregulated miRNAs included miR-106a-5p, miR-106b-5p, miR-17-5p, miR-20a-5p, miR-20b-5p, miR-181b-5p, and miR-128-3p, while miR-127-3p, miR-139-5p, miR-433-3p, and miR-584-5p were downregulated. Validated targets concentrated on key leukemia-related genes like PTEN, BCL2L11, CDKN1A, CCND1, RB1, E2F1, and TGFBR2. KEGG pathway analysis highlighted pathways associated with leukemic cell survival and growth, including MAPK, cell cycle, autophagy, Hippo, ubiquitin-mediated proteolysis, and mTOR signaling pathways. These findings reveal a concise ALL-associated miRNA panel predominantly comprising the miR-17/20/106 family and provide a prioritized set of candidate regulatory networks for subtype-specific validation and functional follow-up studies.

## 1. Introduction

Acute lymphoblastic leukemia (ALL) is the most common pediatric malignancy and a major cause of cancer-related morbidity and mortality in children and adolescents worldwide. Although deaths and disability-adjusted life years (DALYs) attributable to ALL have declined in recent decades, the disease burden remains substantial and heterogeneous across regions and socio-demographic strata, reflecting disparities in timely diagnosis, molecular testing, and access to risk-adapted therapy [1].

Modern ALL management is increasingly anchored in integrated clinicopathologic and molecular classification, aligning with updated frameworks such as the *WHO Classification of Haematolymphoid Tumours (5th edition)*, which emphasizes lineage assignment (B-ALL vs. T-ALL) and recurrent genetic abnormalities to refine diagnosis and prognostication [2]. In current practice, ALL is classified primarily by lineage (B-ALL or T-ALL) and is further stratified by recurrent genetic alterations (e.g., aneuploidy categories and defining gene fusions/rearrangements), which inform risk grouping and therapeutic decisions. The International Consensus Classification (ICC) and WHO5 are broadly concordant in the core diagnostic approach to ALL; differences mainly concern category definitions and nomenclature, and how specific genetic entities are grouped, rather than fundamental changes in the identification of ALL as B- or T-lineage disease [2]. Despite major survival gains, in many settings, relapse, therapy resistance, and treatment-related toxicities remain clinically important challenges. Risk stratification is therefore increasingly dependent on minimal/measurable residual disease (MRD), assessed by multiparameter flow cytometry, PCR-based assays, or next-generation sequencing (NGS), given its strong association with relapse risk and long-term outcomes [3].

Within this landscape, microRNAs (miRNAs), ~18–22-nucleotide non-coding RNAs that post-transcriptionally regulate gene expression, have emerged as biomarker candidates for ALL. miRNAs contribute to normal hematopoiesis and lymphoid differentiation, and their dysregulation may promote leukemogenesis through effects on proliferation, apoptosis, differentiation blockade, and therapy response pathways [4]. Multiple profiling studies support the view that miRNA expression patterns can discriminate ALL from non-leukemic controls and other acute leukemias, while also providing signals related to risk group, MRD, and chemosensitivity [5,6,7]. Importantly, miRNA-based signals are best viewed as complementary to established diagnostic and risk-stratification workflows rather than as stand-alone criteria for ALL diagnosis.

A growing body of evidence suggests that specific miRNAs may serve as diagnostic or discriminatory markers in ALL. Meta-analytic evidence highlights panels (e.g., miR-128a/miR-223) with diagnostic performances and has repeatedly reported miRNAs such as miR-128a/miR-128b, miR-223, let-7b, miR-155, and miR-24 as candidates that help distinguish ALL from AML [8]. More broadly, reviews of ALL miRNA profiling identify recurring candidates at diagnosis, including miR-125b, the miR-128 family, miR-142-3p, miR-155, the miR-181 family, and miR-203 [4].

Beyond diagnosis, miRNAs have been implicated in treatment response and residual disease. For example, circulating miRNAs such as miR-128-3p and miR-222-3p have been reported to decline during induction therapy and correlate with subsequent bone marrow MRD measurements [9]. Other studies associate miRNAs with steroid response and chemoresistance, suggesting that the miRNA-mediated regulation of drug response networks may contribute to inter-patient variability in outcomes [10,11].

However, a key limitation remains: the lack of a universally accepted consensus miRNA signature for ALL, attributable to biological heterogeneity, differences in biospecimen type (bone marrow vs. blood; cellular vs. extracellular fractions), and analytical variability across platforms and pipelines. miRNA datasets underscore that reported differentially expressed miRNAs can cluster by platform rather than disease state while still identifying a subset of repeatedly implicated candidates [12].

Accordingly, this study aimed to identify differentially expressed miRNAs in ALL relative to non-leukemic controls and to perform validated target and pathway analyses to connect dysregulated miRNAs to curated target genes and enriched biological processes relevant to leukemic growth, treatment response, and residual disease.

## 2. Results

### 2.1. Participants’ Results

A total of 24 samples were profiled on the Affymetrix miRNA microarray, comprising 19 cases of acute lymphoblastic leukemia (ALL) and five non-leukemic controls. One ALL sample was excluded from downstream analysis due to exhibiting an aberrant signal distribution in raw-intensity boxplots, based on our predefined exclusion criterion (threshold = 0.15). The control group consisted of five females aged 25–50 years (mean age: 38.8 years). Clinical metadata were linked to 18 ALL profiles. Among the ALL cases, B-ALL was the predominant subtype (16/18), with T-ALL accounting for 2/18. Sex distribution among linked cases was nine females and nine males. Age at diagnosis ranged from 1 to 71 years (median: 6 years; mean: 10.13 years). Initial white blood cell (WBC) counts ranged from 0.9 to 780 (median: 10.34; mean: 86.85) (Table 1). CNS status was recorded as CNS1 in 15/18 and as CNS3 in 3/18. Risk stratification (COG) was available for 16/18 linked cases, with 12/16 classified as high risk and 4/16 as standard risk. End-of-induction marrow response was morphologically M1 in 14 cases and M3 in three cases, with one case not assessed/recorded. Treatment protocols included AALL1131, UKALL relapsed ALL regimens, Interfant protocols, the St Jude protocol, and palliative chemotherapy as documented on the clinical sheet.

### 2.2. Microarray Quality Control and Preprocessing

Microarray and preprocessing were performed before differential expression analysis. Raw array data were quality-assessed for signal distribution, array-to-array variability, and potential outliers using an arrayQualityMetrics-style framework (boxplots, MA plots, distance heatmaps, and PCA). Overall, signal distributions were comparable across arrays, supporting consistent hybridization and normalization performances. One sample was identified as an outlier based on an aberrant signal distribution and was excluded from downstream analyses (Supplementary Figures S1 and S2).

### 2.3. Global Structure of miRNA Expression (PCA/MDS)

**Figure 1:**
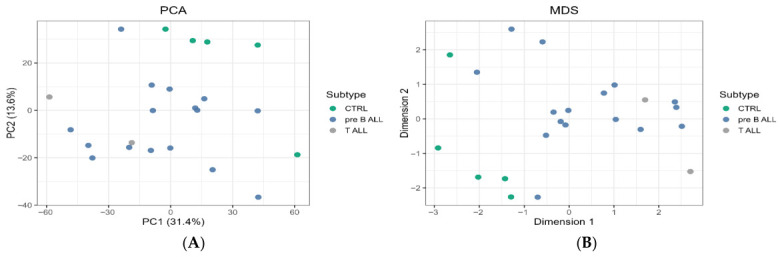
Unsupervised sample-level quality control after preprocessing. (**A**) Principal component analysis (PCA) of miRNA expression profiles following RMA normalization and probe filtering. Samples are colored by subtype (CTRL, pre-B-ALL, T-ALL). PC1 and PC2 explain 31.4% and 13.8% of variance, respectively. The plot shows a trend toward the separation of controls from leukemia samples, with partial overlap among leukemia subtypes. (**B**) Multidimensional scaling (MDS) plot based on pairwise expression distances computed from the postprocessed miRNA expression matrix. Samples cluster primarily by disease status, with controls tending to separate from leukemia cases along Dimension 1, supporting downstream differential expression comparisons. (CTRL: n = 5; pre-B-ALL: n = 16; T-ALL: n = 2). MDS may appear to provide a clearer separation because it is computed from pairwise sample-to-sample distances across the full expression space, whereas PCA reflects the variance captured by the leading components.

Following RMA normalization and filtering, unsupervised ordination was performed. PCA and MDS plots indicated separation between ALL and control samples, consistent with the disease-associated miRNA expression structure (Figure 1).

### 2.4. Differential Expression of miRNAs (ALL vs. Control)

**Figure 2:**
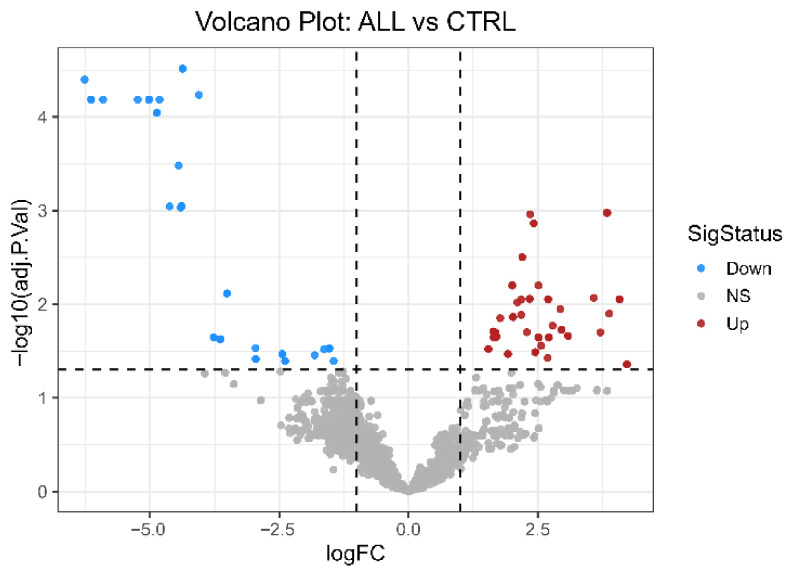
Volcano plot of differentially expressed miRNAs between ALL and healthy controls. Each point represents a miRNA, plotted by log2 fold change (log2FC; ALL vs. CTRL) on the *x*-axis and −log10 of FDR-adjusted *p*-value on the *y*-axis. MiRNAs meeting significance criteria (FDR ≤ 0.05 and |log2FC| ≥ 1) are highlighted in red (upregulated in ALL; log2FC > 0) and blue (downregulated in ALL; log2FC < 0), whereas non-significant miRNAs are shown in gray. Vertical dashed lines indicate fold-change thresholds, and the horizontal dashed line indicates the FDR threshold (CTRL: n = 5, ALL: n = 18; differential expression tested using limma).

Differential expression testing identified sets of dysregulated miRNAs in ALL compared with controls under an FDR framework (FDR ≤ 0.05; |log2FC| ≥ 1). Volcano plots summarize the effect sizes against statistical significance, highlighting miRNAs that are significantly upregulated or downregulated (Figure 2).

### 2.5. Heatmap-Based Visualization of Differential Expression miRNA Signatures

**Figure 3:**
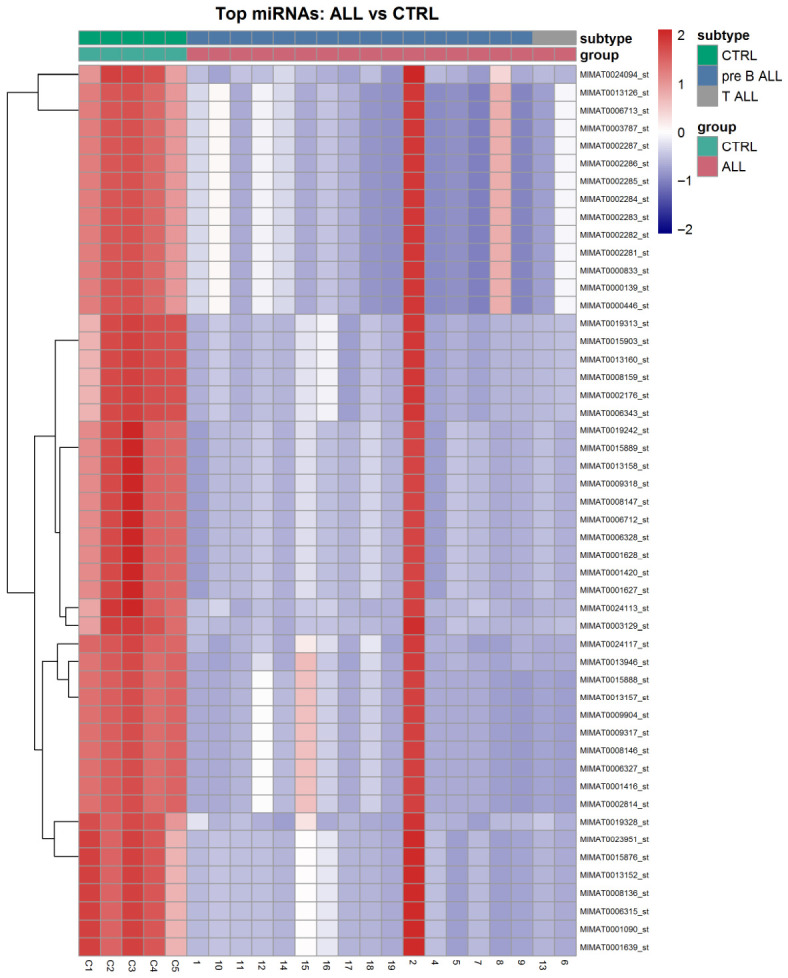
Heatmap of top 50 differentially expressed miRNAs. Row-scaled heatmap showing top 50 miRNAs based on FDR and absolute log2 fold-change. Columns represent individual samples and rows represent miRNAs. Values are standardized within each miRNA (z-score per row), highlighting relative up- and down-expression across samples rather than absolute expression levels. Hierarchical clustering (dendrogram) was applied to miRNA rows; samples (columns) were ordered by disease group and subtype annotation to aid interpretation. Annotation bars indicate sample metadata (e.g., disease group: CTRL vs. ALL; subtype: pre-B-ALL vs. T-ALL), enabling visualization of major global expression patterns and potential group-level structure.

Heatmaps of the top significant miRNAs (ranked by FDR and absolute log2FC) were generated with row-scaled expression and column annotations for the group. Hierarchical clustering revealed distinct expression patterns that differentiated ALL from controls (Figure 3).

### 2.6. Unsupervised Variability Landscape (Top Variable miRNAs)

**Figure 4:**
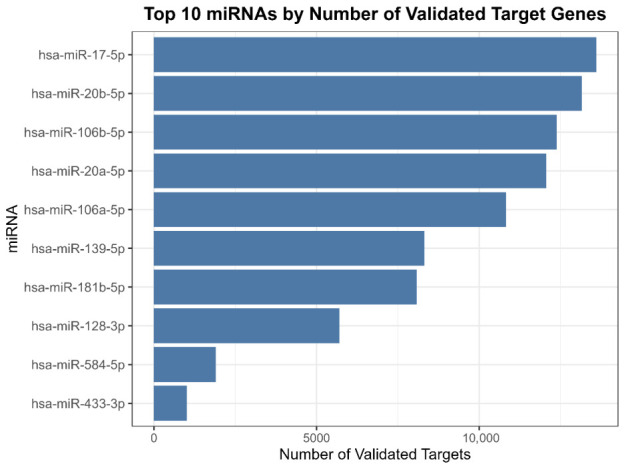
Top 10 miRNAs ranked by number of experimentally validated target genes. Horizontal bar plot showing ten miRNAs with the largest number of experimentally validated target genes retrieved from multiMiR integrating miRTarBase/TarBase. Higher target counts indicate that miRNAs have broad reported regulatory networks, thereby prioritizing candidates for downstream pathway enrichment and mechanistic interpretation in ALL. Target count is used here as a literature-supported prioritization heuristic and does not by itself imply greater clinical severity or pathogenicity.

An unsupervised diagram of the 10 most variable miRNAs across the dataset was generated to highlight the sample-level structure independent of differential testing, including miR-17-5p; miR-20b-5p; miR-106b-5p; miR-20a-5p; miR-106a-5p; miR-139-5p; miR-181b-5p; miR128-3p; miR-584-5p; miR-433-3p (Figure 4).

### 2.7. Validated Target Aggregation Highlights Key Regulatory miRNAs

**Figure 5:**
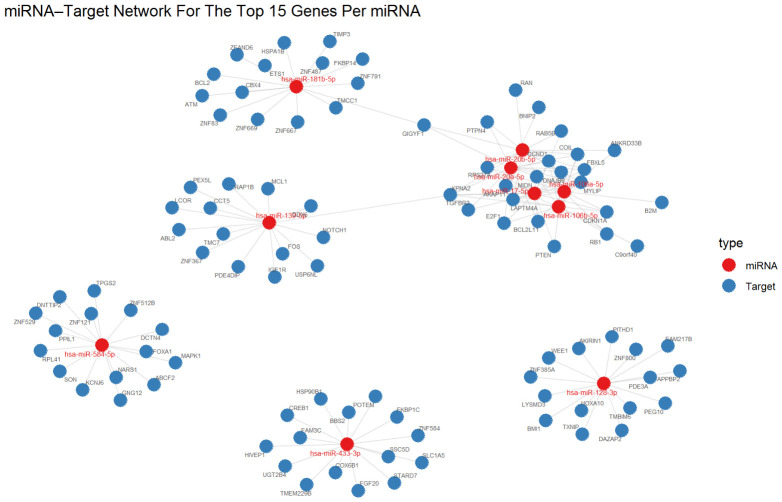
miRNA–target interaction network for prioritized miRNAs in ALL. Network visualization of top 10 miRNAs (red nodes) and their top 15 experimentally validated target genes per miRNA (blue nodes), retrieved from miRTarBase, TarBase, and miRecords. Edges indicate validated miRNA–target interactions. Plot highlights differences in target network breadth across miRNAs and emphasizes shared or hub targets within the validated interaction set. Hub genes include leukemia-relevant nodes such as PTEN, BCL2L11 (BIM), CDKN1A, CCND1, RB1, and E2F1.

An miRNA–target network was generated to explore connectivity patterns between the top dysregulated miRNAs and their validated targets, providing a systems-level representation of validated interactions. Experimentally validated miRNA–target relationships were aggregated. miRNAs were ranked by the number of related validated targets. Unsupervised ranking identified miR-20b-5p, miR-128-3p, miR-652-3p, miR-106a-5p, miR-191-5p, miR-423-5p, miR-142-5p, miR-30c-5p, miR-584-5p, and miR-181b-5p as the most variable miRNAs across samples. Functional enrichment of miRNA target genes using KEGG indicated significant enrichment of the cell cycle pathway, suggesting the altered regulation of the Cyclin D–CDK–RB/E2F axis and cell cycle inhibitory nodes (e.g., CDKN1A/p21), which aligns with the high proliferative behavior of ALL blasts (Figure 5).

### 2.8. Functional Enrichment of Validated Targets (ALL vs. Control)

**Figure 6:**
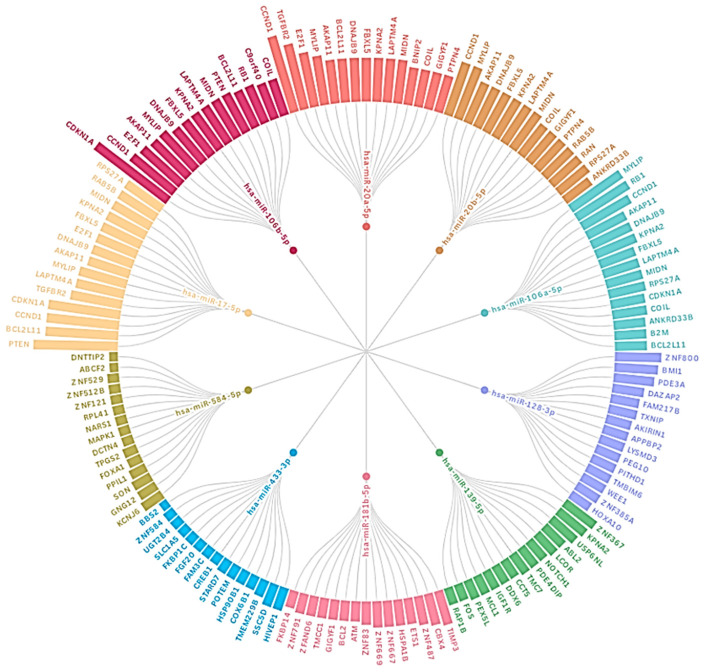
Circular (Circos-style) visualization of experimentally validated miRNA–target interactions for ALL vs. CTRL. Plot summarizes miRNA–gene interactions for prioritized miRNAs identified in ALL vs. CTRL differential expression analysis. miRNAs are shown as inner nodes, and target genes are arranged on the outer ring. Validated interactions were retrieved using multiMiR, restricted to curated validated-source databases (miRTarBase, TarBase, and miRecords), with predicted targets excluded. Top 15 validated targets per miRNA are displayed. Genes that appear under more than one miRNA indicate shared regulatory connections across miRNA set. Edge thickness is proportional to the supporting evidence returned by multiMiR.

Functional enrichment was performed on validated target sets. Gene Ontology (BP/MF/CC) and KEGG pathway enrichment results were summarized using dot plots that report enrichment magnitude and significance (−log10 (FDR)), with dot size indicating gene counts (Figure 6). The top DE miRNAs, dominated by the miR-17/20/106 family, exhibit shared targeting and converge on central leukemia control nodes: PTEN, BCL2L11, CDKN1A, CCND1, RB1, and E2F1. This indicates a coordinated miRNA regulatory program linked to cell cycle progression and survival signaling in ALL, although the functional relevance of these associations requires direct experimental confirmation.

#### 2.8.1. KEGG Crosstalk Matrix Integrating Differential miRNA Expression with Experimentally Validated Targets

**Figure 7:**
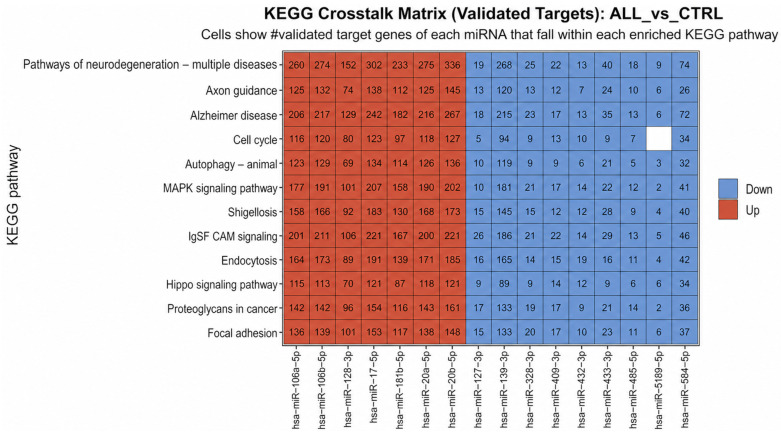
A KEGG crosstalk matrix linking differentially expressed miRNAs to enriched pathways (validated targets; ALL vs. CTRL). A heatmap showing the number of experimentally validated target genes for each differentially expressed miRNA that mapped to each significantly enriched KEGG pathway in the ALL vs. CTRL comparison. The rows represent the enriched KEGG pathways, and the columns represent the differentially expressed miRNAs. Each cell displays the count of validated targets for a given miRNA, annotated to the corresponding KEGG pathway. The column color denotes the direction of miRNA differential expression in the ALL vs. CTRL (red: upregulated; blue: downregulated). This matrix highlights pathway overlap (“crosstalk”). It supports that the miR-17/20/106 family contributes the largest validated set of targets across multiple leukemia-relevant pathways, including cell cycle, MAPK signaling, autophagy, endocytosis, Hippo signaling, and focal adhesion pathways. Some KEGG “disease” pathways can appear due to shared genes with core signaling/trafficking modules.

To connect the differentially expressed miRNAs to biological mechanisms, a KEGG crosstalk matrix was constructed using experimentally validated miRNA–target interactions. The matrix quantifies, for each miRNA, the number of validated target genes that fall within each significantly enriched KEGG pathway. This analysis suggested broad pathway overlap, with the highest target burdens consistently contributed by the upregulated miR-17/20/106 module (miR-17-5p, miR-20a-5p, miR-20b-5p, miR-106a-5p, and miR-106b-5p). These hub miRNAs collectively mapped to numerous pathway genes across leukemia-relevant programs, including cell cycle, MAPK signaling, autophagy, endocytosis, Hippo signaling, and microenvironment-associated categories such as focal adhesion and proteoglycans in cancer. In contrast, downregulated miRNAs showed comparatively fewer validated targets per pathway. Overall, the crosstalk matrix supports a network model in which a small subset of highly connected miRNAs accounts for much of the pathway-level enrichment observed in ALL versus controls (Figure 7).

#### 2.8.2. GO and KEGG Enrichment of Validated Targets

**Figure 8:**
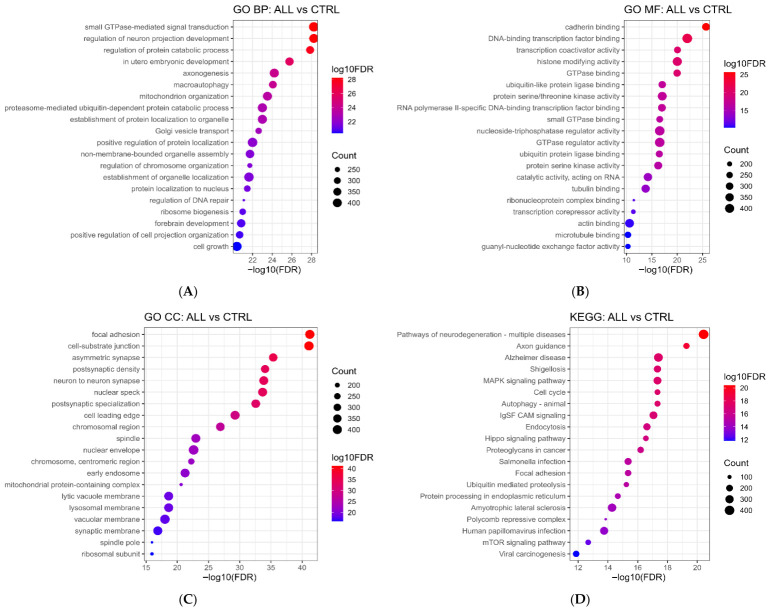
Dot plots summarizing enrichment results for experimentally validated target genes of significantly differentially expressed miRNAs. Three Gene Ontologies in the next GO term are presented on the *x*-axis, while the number and percentage of target gene candidates annotated in this GO term are presented on the *y*-axis. (**A**) GO Biological Process Enrichment log10 (FDR) GO Biological Process (BP) terms enriched in validated target genes of significantly dysregulated miRNAs are displayed in a dot plot. The number of genes is represented by dot size, and −log10 (FDR) is represented by color. Stronger enrichment is indicated by higher values. (**B**) GO Molecular Function Enrichment (log10FDR) Enriched GO Molecular Function (MF) terms for validated miRNA targets. Dot size corresponds to gene count; color corresponds to −log10 (FDR). Pathways represent functional processes altered in the examined leukemia subtype. (**C**) GO Cellular Component Enrichment (log10FDR) Dot plot for validated miRNA targets displaying enriched Cellular Component (CC) terms. Greater representation in gene sets and pathways with greater significance is indicated by larger/redder dots. (**D**) KEGG Pathway Enrichment log10 (FDR) dot plot of enriched KEGG pathways based on validated miRNA targets. Dot size reflects number of target genes involved; dot color represents −log10 (FDR). Significantly enriched pathways indicate downstream biological mechanisms influenced by dysregulated miRNAs.

Functional enrichment analysis was performed on the experimentally validated target genes of the significantly dysregulated miRNAs in the ALL vs. CTRL comparison (Figure 8). Across Gene Ontology categories, GO Biological Process (BP) enrichment showed a strong overrepresentation of processes related to small GTPase-mediated signal transduction, regulation of neuron projection development/axonogenesis, protein catabolic processes, organelle organization and localization, Golgi vesicle transport, and proteasome/ubiquitin-dependent protein catabolism, with high significance indicated by elevated −log10 (FDR) values and large gene counts (Figure 8A).

In the GO Molecular Function (MF), enriched terms were dominated by binding and regulatory activities, including cadherin binding, DNA-binding transcription factor binding, transcription cofactor/corepressor activity, histone-modifying activity, ubiquitin(-like) protein ligase binding, protein serine/threonine kinase activity, and multiple GTPase regulator/binding functions (Figure 8B).

For the GO Cellular Component (CC), the validated target set was significantly enriched for subcellular localizations linked to cellular interaction and structural organization, including focal adhesions, cell–substrate junctions, synaptic/post-synaptic compartments, nuclear specks and nuclear envelopes, as well as membrane and trafficking-related compartments, such as early endosome and lysosomal/vacuolar membranes and the mitochondrial protein-containing complex (Figure 8C).

Consistent with these GO results, KEGG pathway enrichment analysis identified the significant overrepresentation of pathways related to MAPK signaling, the cell cycle, autophagy, endocytosis, Hippo signaling, IgSF CAM signaling, and focal adhesion (Figure 8D). Across panels, dot size reflects the number of validated target genes assigned to each term/pathway, while dot color and *x*-axis position (−log10 FDR) indicate enrichment significance.

### 2.9. Clinical Association Checks

**Figure 9:**
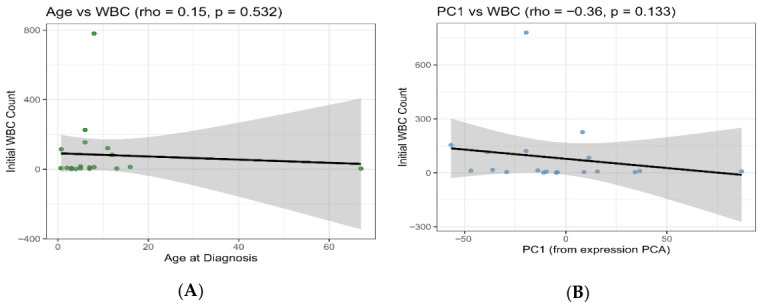
Associations between the clinical variables and initial WBC count. (**A**) A scatter plot of age at diagnosis vs. initial white blood cell (WBC) count. The black line indicates the fitted trend, and the shaded region shows the 95% confidence interval. The Spearman’s rank correlation (ρ) and *p*-value are reported in the panel title. (**B**) A scatter plot of the first principal component (PC1) from the miRNA expression PCA vs. initial WBC count, with the fitted trend line and 95% confidence interval. The Spearman’s ρ and *p*-value are shown in the title. In this cohort, the observed correlations are not statistically significant, suggesting no strong monotonic association between the WBC and either age or global variation summarized by PC1.

Exploratory correlations were performed to examine associations between the clinical variables and expression-derived features, including the age at diagnosis versus baseline WBC count and PC1 versus baseline WBC count, using Spearman correlation. The initial WBC count was not significantly correlated with the age at diagnosis (Spearman ρ = 0.15, *p* = 0.532), and showed only a non-significant negative trend with the first principal component (PC1) of the miRNA expression (ρ = −0.36, *p* = 0.133). These findings suggest that, in this cohort, presenting leukocytosis is not a major driver of the dominant miRNA expression axis, indicating that the observed miRNA signature primarily reflects disease- and subtype-associated regulatory programs rather than simply variation in the initial WBC (Figure 9).

**Figure 10:**
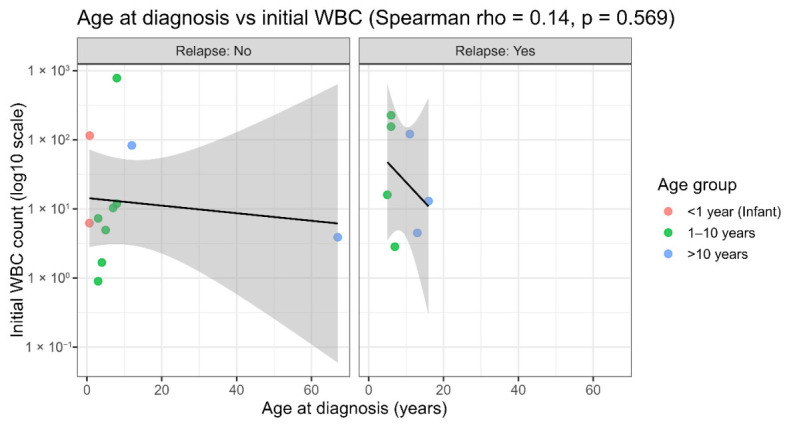
Scatter plots of (age vs. WBC stratified by relapse). Relationship between age at diagnosis and initial WBC count stratified by relapse status. Scatter plots show age at diagnosis (years) versus initial white blood cell (WBC) count plotted on log10 scale, displayed separately for patients with no relapse (**left**) and relapse (**right**). Each point represents one patient and is colored by age group: <1 year (infant), 1–10 years, and >10 years. Solid black line indicates fitted trend with 95% confidence interval (shaded gray). Overall association was assessed using Spearman’s rank correlation (ρ = 0.14, *p* = 0.569), indicating no significant correlation between age at diagnosis and initial WBC count in this cohort.

**Figure 11:**
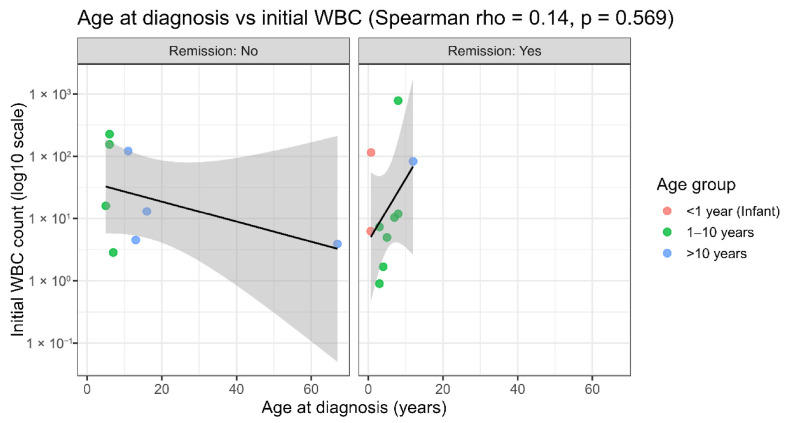
Scatter plots of (age vs. WBC stratified by remission). Association between age at diagnosis and initial WBC count stratified by remission status. Scatter plots show age at diagnosis (years) versus initial white blood cell (WBC) count displayed on log10 scale, presented separately for patients who did not achieve remission (**left**; remission: no) and those who achieved remission (**right**; remission: yes). Each dot represents one patient and is colored by age group: <1 year (infant), 1–10 years, and >10 years. A solid black line indicates the fitted trend with a 95% confidence interval (shaded gray). Overall relationship was evaluated using Spearman’s rank correlation (ρ = 0.14, *p* = 0.569), indicating no significant monotonic association between age at diagnosis and initial WBC count in this cohort.

The relationship between the age at diagnosis and baseline WBC count was displayed separately for patients with and without relapse, showing no clear monotonic association within either stratum and supporting the overall weak correlation observed across the cohort (Figure 10). The same relationship was observed for remission status (remission achieved vs. not achieved), supporting the broad dispersion of WBC values across the age range and the absence of a consistent trend within outcome groups (Figure 11). Collectively, these outcome-stratified visualizations indicate that the lack of association between age and the presenting WBC is not driven by a single outcome subgroup, and they further support the interpretation that baseline leukocytosis is unlikely to confound the major miRNA expression patterns detected in this study.

## 3. Discussion

This study identifies a compact miRNA program that differentiates acute lymphoblastic leukemia (ALL) from non-leukemic controls and converges on core oncogenic processes. The heatmap of the top differentially expressed miRNAs shows coherent sample stratification (ALL vs. CTRL) with an additional structure consistent with disease heterogeneity, supporting a reproducible miRNA signature rather than sporadic, sample-specific variation. In parallel, prioritization based on the experimentally validated target burden and network connectivity highlights a small subset of miRNAs with disproportionate regulatory reach, suggesting that ALL-associated miRNA dysregulation is organized around these miRNAs, which can coordinate multi-pathway remodeling. This study’s contribution is the integration of PBMC-derived miRNA profiling with a validated-target-only analysis (multiMiR restricted to curated validation sources), enabling network and pathway interpretation on a clinically annotated cohort.

The global separation of ALL versus controls in the heatmap and ordination plots supports that downstream differential and network analyses are performed on a consistent disease-associated signal rather than being driven by a small number of outliers [13].

A major signal in the prioritized set is the enrichment of the miR-17/20/106 family, including miR-17-5p, miR-20a-5p, miR-20b-5p, miR-106a-5p, and miR-106b-5p, a group repeatedly implicated in lymphoid transformation and treatment resistance. Functionally, this module is known to promote proliferation while buffering apoptosis, in part by repressing key tumor suppressor and pro-apoptotic nodes [14]. The miR-17~92 axis modulates survival control by regulating the BCL2 family and can inhibit BIM-driven apoptosis, thereby supporting leukemic expansion [15,16,17]. Importantly, experimental work in pediatric T-ALL has demonstrated that miRNAs from the related miR-106a-363 cluster, including miR-20b-5p, can suppress apoptosis. Additionally, it can promote tumor suppressor circuitry, including PTEN/BIM-related survival control, consistent with an oncomiR role for this subnetwork in ALL [18]. Our interaction network reinforces this interpretation by placing canonical growth and survival genes among shared, high-connectivity targets. These pathways include cell cycle progression (CCND1), checkpoint restraint (CDKN1A/p21), and G1/S control (RB1/E2F1). Other pathways involved in the regulation of proliferation and apoptosis are the PI3K brake (PTEN) and (BCL2L11/BIM). These targets provide a biologically plausible link between the observed miRNA signature and the KEGG enrichment for the cell cycle and MAPK signaling pathways. Collectively, these findings are consistent with the possibility that dysregulated miRNAs in ALL may contribute to proliferative and survival-associated programs; however, direct mechanistic confirmation was beyond the scope of the present study.

To strengthen translational confidence, the top differentially expressed miRNAs identified by microarray require independent confirmation by qRT-PCR, ideally in the same samples and in an independent cohort. Likewise, direct functional testing of selected miRNA–target edges (e.g., PTEN, BCL2L11, and CDKN1A) using reporter-based or perturbation assays would be necessary to confirm the mechanistic relationships proposed here. Because these additional experiments were not feasible within the current revision, the present findings should be interpreted as discovery-oriented and hypothesis-generating. Accordingly, the network and pathway analyses are intended to prioritize candidate miRNAs and regulatory interactions for future validation rather than to establish direct causality in this cohort.

### 3.1. Disease-Relevant miRNAs in Lineage Programs and Tumor Suppressor Restraint

miR-17/20/106 and miR-128-3p have strong leukemia-specific evidence as oncomiRs in T-ALL and can accelerate leukemia development in vivo and suppress the tumor suppressor PHF6, linking them directly to T-ALL pathogenesis [19]. miR-181b-5p has been associated with ALL molecular diversity and therapeutic response; earlier large studies connected miR-181 expression patterns to drug resistance phenotypes and prognosis in childhood ALL, supporting our findings in this area [20]. Also, miR-139-5p is widely described as a tumor suppressor miRNA in acute myeloid leukemia (AML), and its downregulation has been linked to enhanced leukemic fitness [21]. However, many core cancer control processes are shared between ALL and AML [22,23]. Thus, the directionality of its function when it is downregulated makes it a possible restraining node in hematologic malignancy networks [24]. miR-433-3p and miR-584-5p were identified as tumor suppressors; however, miR-584-5p was found to act as both a tumor suppressor and an oncogene [25].

### 3.2. Network Structure Indicates Convergence on Shared Regulatory Choke Points

The miRNA target network and Circos visualization show that multiple prioritized miRNAs converge on overlapping targets, suggesting the redundant repression of key checkpoints, including CCND1/CDKN1A/RB1. This architecture is important in leukemia because it can provide the partial inhibition of one miRNA, which may be buffered by another miRNA targeting the same node. The presence of shared targets involved in transport/protein homeostasis, such as KPNA2 and ubiquitin-related genes, also supports the idea that miRNA dysregulation in ALL is not limited to proliferation/apoptosis but extends to cellular functions such as trafficking, turnover, and stress handling, thereby influencing drug response.

The KEGG crosstalk matrix, integrating differential miRNA expression with experimentally validated targets, suggested a supporting direction for the Circos visualization. The up- and downregulated miRNAs with their corresponding KEGG pathways, a group of upregulated miRNAs largely comprising the miR-17/20/106 family (miR-17-5p, miR-20a-5p, miR-20b-5p, miR-106a-5p, miR-106b-5p), together with miR-128-3p and miR-181b-5p, showed the highest validated target coverage across all enriched pathways, including the cell cycle, MAPK signaling, autophagy, endocytosis, Hippo signaling, focal adhesion, and proteoglycans in cancer. In contrast, the set of downregulated miRNAs (including miR-127-3p, miR-139-5p, miR-433-3p, and miR-584-5p) mapped to comparatively fewer validated targets per pathway. Collectively, these findings support a model in which ALL-associated miRNA dysregulation is organized around an upregulated hub module with broad pathway reach, accompanied by downregulated miRNAs that may reflect reduced tumor-suppressive buffering.

### 3.3. KEGG Results Highlight Several Pathway Classes That Are Highly Plausible in ALL Pathophysiology

The KEGG cell cycle pathway enrichment suggests dysregulation of the G1/S transition and checkpoint control, involving the Cyclin D–CDK–RB1/E2F axis and CDK inhibitors such as CDKN1A (p21) [26,27]. This is consistent with the hyperproliferative biology of acute lymphoblastic leukemia. MAPK signaling was significantly enriched among the validated targets of dysregulated miRNAs, identifying that these miRNAs may modulate key growth and stress response signaling nodes relevant to proliferation and survival in ALL. The PI3K/AKT/mTOR axis is functionally implicated through PTEN and is upregulated in ALL [28]. This upregulation is associated with a poor prognosis and chemoresistance, making it a credible downstream consequence of the miRNA group suppression of inhibitory nodes [28]. Endocytosis regulates receptor recycling and niche signaling. Endocytosis has been shown to increase leukemia stem cell signaling and chemoresistance, and its inhibition can impair multiple niche-derived survival cues [29]. This aligns with the idea that miRNA programs may cooperate with trafficking pathways to stabilize survival signaling in the marrow microenvironment. Autophagy can be pro-survival in leukemic cells under metabolic and therapeutic stress, and targeting autophagy has been proposed as a therapeutic strategy in pediatric ALL, including sensitization contexts [30]. Autophagy enrichment, therefore, supports a stress adaptation component within the miRNA–target landscape. Also, adhesion pathways are directly connected to ALL persistence through bone marrow and the CNS microenvironment. Adhesion has been reported to contribute to survival and chemoresistance [31]. The appearance of focal adhesion terms in the KEGG is therefore consistent with a model in which miRNA dysregulation contributes to intrinsic proliferation and microenvironmental stability [32]. Enrichment of ubiquitin-mediated proteolysis and ER processing is linked to protein quality control and turnover, which are regulated layers in ALL. This is relevant because proteostasis regulates apoptosis and the availability of signaling proteins, and targeting the ubiquitin pathway is involved in lymphoid malignancies [33]. Although the pathways of “neurodegeneration/Alzheimer/axon guidance” were detected among the identified pathways, this can be interpreted in leukemia due to the shared expression of molecules involved in adhesion, guidance cues, and vesicle trafficking [34]. As a complementary view, future analyses will extend enrichment to oncogenic signature collections (e.g., MSigDB Hallmark and Reactome) to assess whether the validated target set recapitulates broader cancer pathway programs.

### 3.4. Clinical Correlation Checks Support Minimal Confounding by Baseline Leukocytosis

In this cohort, baseline leukocytosis showed no statistically significant association with age at diagnosis and did not align strongly with the dominant axis of miRNA expression variation (PC1). The weak, non-significant correlation between age and initial WBC suggests that presenting leukocytosis is not systematically driven by age in this dataset, despite known age-related differences in ALL biology and prognosis and age being a core clinical stratifier in ALL risk classification [35]. Similarly, the non-significant negative trend between the initial WBC and PC1 is consistent with the fact that the primary miRNA expression gradient captured by PCA is unlikely to reflect simple differences in the tumor burden or peripheral blood cell counts at presentation. This interpretation is strengthened by evidence that bulk PBMC-derived expression signals can be driven by underlying cell-type proportions and cellular heterogeneity rather than a single clinical parameter and therefore require careful covariate evaluation [36]. Collectively, these findings support the interpretation that the major miRNA signal detected in this study is more consistent with disease-associated and subtype-related regulatory programs than with variation in the baseline WBC.

Outcome-stratified visualizations further reinforce this conclusion. When the age–WBC relationship was examined separately by relapse status (Figure 10) and remission status (Figure 11), no clear monotonic trends emerged within either subgroup. This is consistent with the fact that the overall lack of association is not driven by a single clinical outcome category. This is relevant because relapse and failure to achieve remission/induction failure represent clinically high-risk disease states and are closely linked to treatment resistance and inferior outcomes in contemporary ALL trials and treatment frameworks. If the baseline WBC were a dominant confounder of the observed miRNA patterns, a more consistent relationship might be expected within outcome-defined strata. Instead, the broad dispersion of WBC values across ages and outcomes suggests that baseline leukocytosis alone is insufficient to explain the principal structure of miRNA expression.

These correlation checks do not exclude more complex relationships, including non-linear effects, interactions with immunophenotype/genetic subtypes, or shifts in leukemic blast fractions within PBMC preparations. Additionally, the modest sample size may limit the power to detect small-to-moderate effects. Nevertheless, the convergent evidence across the overall cohort and outcome-stratified analyses provides supportive context that the baseline WBC is unlikely to be a major driver of the key miRNA expression signature reported here. Future analyses in larger cohorts with complete clinical annotation could test multi-variable models that jointly account for age, WBC, subtype, and treatment response to better delineate independent clinical and biological contributors to miRNA dysregulation in ALL.

## 4. Materials and Methods

### 4.1. Patient Samples and Metadata

Clinical specimens consisted of peripheral blood mononuclear cells (PBMCs) isolated from peripheral blood collected in anticoagulant tubes (EDTA or heparin). Samples were assigned to biologically defined groups: acute lymphoblastic leukemia (ALL; n = 24) and non-leukemic controls (n = 5). Where available, ALL cases were further annotated by lineage (B-ALL or T-ALL) and available subtype information (Table 1). Inclusion criteria for ALL comprised a confirmed diagnosis of ALL based on routine clinical and laboratory assessment at KAUH and the availability of adequate PBMC material/RNA for downstream profiling. Exclusion criteria included an insufficient sample quantity/quality, receipt of chemotherapy at the time of sampling, and use of anti-inflammatory drugs. Control PBMCs were obtained from individuals undergoing routine blood testing for non-malignant indications with no history of hematologic malignancy and were selected to be as comparable as possible to the ALL cohort with respect to the available demographic variables (Table 1). A structured sample sheet linked each sample to the phenotype variables (group, sample ID) and available clinical covariates (e.g., age at sampling/diagnosis, baseline white blood cell count), obtained from the Department of Laboratory Medicine and Pathology at King Abdulaziz University Hospital (KAUH).

All procedures were conducted under Institutional Review Board approval (IRB No. 512-21, Unit of Biomedical Ethics, King Abdulaziz University) and in accordance with applicable ethical regulations. Written informed consent was obtained from all participants; for minors, consent was obtained from a parent/legal guardian (and assent when required by the IRB).

### 4.2. PBMC Isolation

PBMCs were isolated from peripheral whole blood using a standard density gradient method. Briefly, venous blood collected into anticoagulant tubes (EDTA or heparin) was processed at room temperature within 1–2 h of collection. Blood was diluted 1:1 with sterile phosphate-buffered saline (PBS) and carefully layered onto Ficoll-Paque PLUS (GE Healthcare, Chicago, IL, USA) in 15- or 50-mL conical tubes. Samples were centrifuged at 400–800× *g* for 20–30 min with the brake off to preserve layer separation. The PBMC layer at the plasma–Ficoll interface was aspirated, transferred to a fresh tube, and washed twice with PBS (centrifugation: 300–400× *g* for 8–10 min) to remove platelets and residual Ficoll. The final PBMC pellet was resuspended in PBS. Cell count and viability were assessed using trypan blue exclusion and a hemocytometer or an automated cell counter. For RNA work, PBMCs were pelleted and either lysed immediately in the appropriate lysis buffer or stored at −80 °C until RNA extraction. All reagents and consumables were RNase-free. Samples from ALL and control groups were processed using the same protocol.

### 4.3. RNA Extraction and Quality Assessment

Total RNA was extracted from isolated PBMCs using the RNeasy Mini Kit (Qiagen, Hilden, Germany) according to the manufacturer’s instructions, using RNase-free consumables and water. The RNA concentration and purity were assessed using a NanoDrop 2000c spectrophotometer (Thermo Fisher Scientific, Waltham, MA, USA), and samples with A260/280 ratios of approximately 2.0 were considered acceptable. For microarray labeling, 130 ng of total RNA was used as input.

### 4.4. Affymetrix miRNA Microarray Profiling

Samples meeting internal quality thresholds proceeded to labeling and hybridization. A brief tailing reaction was performed, followed by ligation of the biotinylated signal molecule to the target RNA sample and labeling using the Affymetrix FlashTag Biotin HSR RNA Labeling Kit (Santa Clara, CA, USA). Labeled RNA was hybridized to the Affymetrix GeneChip miRNA 4.0 array (Santa Clara, CA, USA). Arrays were incubated at 48 °C and 60 rpm for 16–18 h using a GeneChip^®^ Hybridization Oven 645 (Santa Clara, CA, USA). Washing and staining were performed on a GeneChip™ Fluidics Station 450 (Santa Clara, CA, USA) according to the manufacturer’s protocol, and arrays were scanned using the GeneChip Scanner 3000 7G to generate CEL files for downstream analysis. Standard Affymetrix/AGCC (or equivalent) software pipelines (Transcriptome Analysis Console (TAC) version 4.0) were used for image acquisition and initial feature extraction.

### 4.5. Data Preprocessing and Normalization

All bioinformatics analyses were conducted in R (version 4.4.2) using Bioconductor (version 3.19) packages. Raw CEL files were imported using the appropriate Affymetrix array-handling package (oligo), with affy used if required for platform compatibility. Expression values were normalized using the Robust Multi-Array Average (RMA), producing log2-transformed expression estimates for each miRNA probe set.

Array-level quality control (QC) was assessed using standard diagnostic visualizations, including raw-intensity distribution boxplots, MA plots, distance-based metrics, and ordination methods, such as principal component analysis (PCA). Automated QC reports were generated using the arrayQualityMetrics package (v3.62.0) to support the systematic inspection of the sample quality. One sample exhibiting an aberrant signal distribution in the raw-intensity boxplots was excluded from downstream analyses based on a predefined exclusion criterion (threshold = 0.15).

### 4.6. Exploratory Data Analysis

Unsupervised analyses were performed on normalized data to evaluate the global structure. Principal component analysis (PCA) and multidimensional scaling (MDS) were used to assess separation between ALL and controls. Heatmaps were generated for the top differentially expressed miRNAs (e.g., the top 50 by FDR and |log2FC|) to visualize specific patterns and sample-to-sample similarity.

### 4.7. Differential Expression (DE) Analysis

Differential expression was computed using the linear modeling of microarray data. A design matrix was constructed to encode group membership. Differential expression was estimated using empirical Bayes-modulated statistics (limma version 3.62.2), which improves the variance estimation in small-to-moderate sample sizes. A single prespecified contrast comparing ALL versus non-leukemic controls was tested. The primary DE criterion applied a false discovery rate (FDR) control using the Benjamini–Hochberg procedure (FDR ≤ 0.05) and a minimum effect size threshold (|log2FC| ≥ 1). Volcano plots (log2FC vs. −log10 adjusted *p*-values) and heatmaps of the top DE miRNAs were produced for each contrast. Full result tables (miRNA ID, log2FC, standard error, *p*-value, adjusted *p*-value) were exported for reproducibility.

### 4.8. miRNA Target Gene Retrieval (Experimentally Validated Interactions)

Experimentally validated miRNA–target interactions were retrieved using the multiMiR package (version 1.28.0). Queries were restricted to validated interactions only (table = “validated”), excluding all predicted targets. Validated interactions were sourced from curated databases, including miRTarBase (v9), TarBase (v9), and miRecords (v4). For each significant miRNA set (by contrast), validated targets were aggregated and summarized (e.g., number of validated targets per miRNA), and the top miRNAs by validated target count were highlighted as candidate regulatory hubs.

### 4.9. Functional Enrichment Analysis of Validated Targets

Validated target genes were subjected to functional enrichment to identify overrepresented biological programs. Gene Ontology enrichment was conducted for Biological Processes (BPs), Molecular Functions (MFs), and Cellular Components (CCs). KEGG pathway enrichment analysis was performed on validated miRNA target genes using the enrichKEGG function from the clusterProfiler package (clusterProfiler_4.14.6). Pathways with a false discovery rate (FDR) ≤ 0.05 were considered significantly enriched and visualized as dot plots showing gene counts and −log10 (FDR) values.

### 4.10. miRNA–Target Network Visualization

To present regulatory relationships, miRNA–target networks were summarized using the top 10 most informative miRNAs and their top 15 validated targets per miRNA (selected based on interaction support and target burden ranking). Network outputs were generated where appropriate to facilitate the exploratory inspection of connectivity patterns.

### 4.11. Exploratory Clinical Association Analyses

Exploratory analyses were performed to examine associations between the clinical variables and expression-derived features. Spearman’s rank correlation was used to assess relationships between age at diagnosis, initial white blood cell (WBC) count, and first principal component (PC1) of miRNA expression. Scatter plots with linear smoothing were generated, and correlation coefficients and corresponding *p*-values were reported. These analyses were exploratory and intended to identify potential associations.

## 5. Conclusions

In summary, PBMC-derived miRNA profiling identified an ALL-associated miRNA signature dominated by the miR-17/20/106 family and miR-128-3p, with experimentally validated target databases indicating convergence on cell cycle- and survival-related pathways. These findings define a focused set of candidate miRNAs and regulatory networks for subtype-specific follow-up studies. Because qRT-PCR did not independently confirm the microarray results and no direct functional validation of miRNA–target interactions was performed in the present cohort, the current study should be regarded as discovery-oriented and hypothesis-generating. Therefore, the biological and translational implications of these findings require confirmation in larger independent cohorts and by targeted functional assays.

The pathway findings can motivate therapeutic hypotheses such as the combined targeting of PI3K/AKT/mTOR, endocytosis, or autophagy in miRNA-defined subgroups. Finally, the microarray findings were not independently confirmed by qRT-PCR in this study, and confirmatory validation is warranted.

Like other studies, this work has several limitations. First, the cohort size was modest (n = 23), which limits the statistical power, reduces the ability to perform robust subtype-stratified analyses, and may affect the generalizability of the findings. Second, there was a marked age imbalance between groups: the controls were adults (mean ± SEM: 35 ± 3 years), whereas the ALL group consisted predominantly of pediatric patients (mean age: 12.1 years). This imbalance reflects the practical difficulty of recruiting truly healthy pediatric controls, as blood collection from non-hospitalized children is ethically and logistically challenging. Consequently, age-related differences in circulating miRNA profiles cannot be fully excluded as a potential confounder. Third, because pediatric samples were collected from hospitalized patients, it was not possible to completely rule out concurrent conditions (e.g., infections, inflammation, or other comorbidities) that might have influenced the miRNA expression independent of ALL. Fourth, key clinical variables, such as MRD status at the end of induction, cytogenetic and molecular alterations, pre- versus post-therapy biology, remission status, and relapse status, were not included in the analysis and may have influenced the findings. Fifth, the microarray-based differential expression findings were not independently validated by qRT-PCR, and the proposed miRNA–target relationships were inferred from curated, experimentally validated databases rather than directly tested in our own samples; therefore, causal and translational interpretations should be made with caution.

Future studies should include larger age-matched control groups and independent validation cohorts, and, where feasible, incorporate additional clinical covariates to eliminate residual confounding.

## Figures and Tables

**Table 1:** Summary of participant characteristics and available clinical metadata.

Characteristic	ALL	Controls
Microarray profiles (n)	19 (18 after QC exclusion)	5
Sex	9 F/9 M (linked cases)	5 F
Age (years)	Range: 1–71; median: 6; mean: 10.13	Range: 25–50; mean: 38.8
Subtype (linked cases)	B-ALL: 16/18; T-ALL: 2/18	NA
Initial WBC count	Range: 0.9–780; median: 10.34; mean: 86.85	NA
CNS status (linked cases)	CNS1: 15/18; CNS3: 3/18	NA
COG risk (available cases)	High: 12/16; standard: 4/16	NA
End-of-induction marrow (available cases)	M1: 14; M3: 3; 1 not recorded	NA
Treatment protocols (as recorded)	AALL1131, UKALL relapsed regimens, Interfant, St Jude, palliative	NA

## Data Availability

All relevant data are within the manuscript and its Supporting Materials Files. The datasets generated and/or analyzed during the current study are available in the NCBI Gene Expression Omnibus (GEO) repository under accession number GSE322536 https://www.ncbi.nlm.nih.gov/geo/query/acc.cgi?acc=GSE322536, accessed on 2 March 2026.

## Supplementary Materials

The following supporting information can be downloaded at: https://www.mdpi.com/article/10.3390/ijms27093868/s1.

## Abbreviations

The following abbreviations are used in this manuscript:

AGCC	Affymetrix GeneChip Command Console
ALL	Acute lymphoblastic leukemia
AML	Acute myeloid leukemia
B-ALL	B-cell acute lymphoblastic leukemia
BCL2L11 (BIM)	BCL2-like 11
BH	Benjamini–Hochberg
BP	Biological process
CC	Cellular component
CCND1	Cyclin D1
CDKN1A (p21)	Cyclin-dependent kinase inhibitor 1A
CEL files	Cell intensity files generated from Affymetrix arrays
CNS	Central nervous system
COG	Children’s Oncology Group
CTRL	Control
DALYs	Disability-adjusted life years
DE	Differential expression
DNA	Deoxyribonucleic acid
E2F1	E2F transcription factor 1
EDTA	Ethylenediaminetetraacetic acid
FDR	False discovery rate
GEO	Gene Expression Omnibus
GO	Gene Ontology
IRB	Institutional Review Board
KEGG	Kyoto Encyclopedia of Genes and Genomes
limma	Linear models for microarray data
log2FC	Log2 fold change
MAPK	Mitogen-activated protein kinase
MDS	Multidimensional scaling
MF	Molecular function
miRNA	MicroRNA
miRecords	MicroRNA Records database
miRTarBase	MicroRNA Target Database
mRNA	Messenger ribonucleic acid
MRD	Minimal residual disease
mTOR	Mechanistic target of rapamycin
multiMiR	Multidatabase miRNA–target interaction resource
NCBI	National Center for Biotechnology Information
NGS	Next-generation sequencing
PBMCs	Peripheral blood mononuclear cells
PCA	Principal component analysis
PC1	First principal component
PCR	Polymerase chain reaction
PBS	Phosphate-buffered saline
PHF6	PHD finger protein 6
PI3K	Phosphoinositide 3-kinase
PTEN	Phosphatase and tensin homolog
QC	Quality control
RB1	Retinoblastoma 1
RMA	Robust multi-array average
RNA	Ribonucleic acid
rpm	Revolutions per minute
SEM	Standard error of the mean
TarBase	TarBase database of experimentally supported miRNA targets
T-ALL	T-cell acute lymphoblastic leukemia
TGFBR2	Transforming growth factor beta receptor 2
WBC	White blood cell
WHO	World Health Organization
