# Supplementary material for: Differential Expression and Target Gene Analysis of PBMC-Derived microRNAs as Prognostic Biomarkers in Acute Lymphoblastic Leukemia
Source: Int J Mol Sci. 2026 Apr 27;27(9):3868. doi: 10.3390/ijms27093868 (PMC13163416; doi:10.3390/ijms27093868)
Supplement: Supplementary file 1 [file ijms-27-03868-s001.zip › ijms-4233275-Supplementary Materials.pdf]

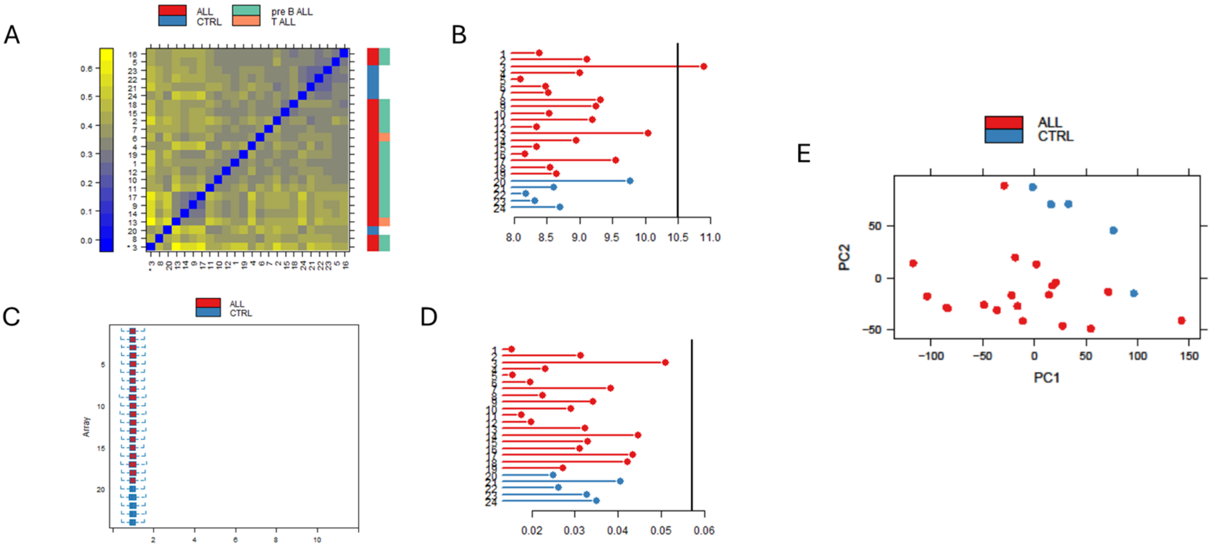

Figure S1. Quality metrics plots before exclusion (n = 24). (**A**) A distance heatmap between arrays. The heatmap compares each array to all others using the L1 distance. According to the plot, one array was flagged as an outlier because it had a large total distance to all other arrays. The result shows that one array exceeded the threshold of 10.5 and was considered an outlier. (**B**) Outlier detection shows the exact bar that caused the outlier call. Threshold = 10.5. One array crosses it. (**C**) Boxplots of raw intensities. The boxplots are mostly aligned. No arrays were considered outliers based on the intensity distribution (Kolmogorov–Smirnov test). All boxes sit on the same level, indicating a uniform signal with no arrays flagged. (**D**) Outlier detection from boxplots. Threshold = 0.0572 (none exceeded it). (**E**) PCA of arrays, showing PC1 vs. PC2 using raw data. The points cluster, indicating a consistent overall structure.

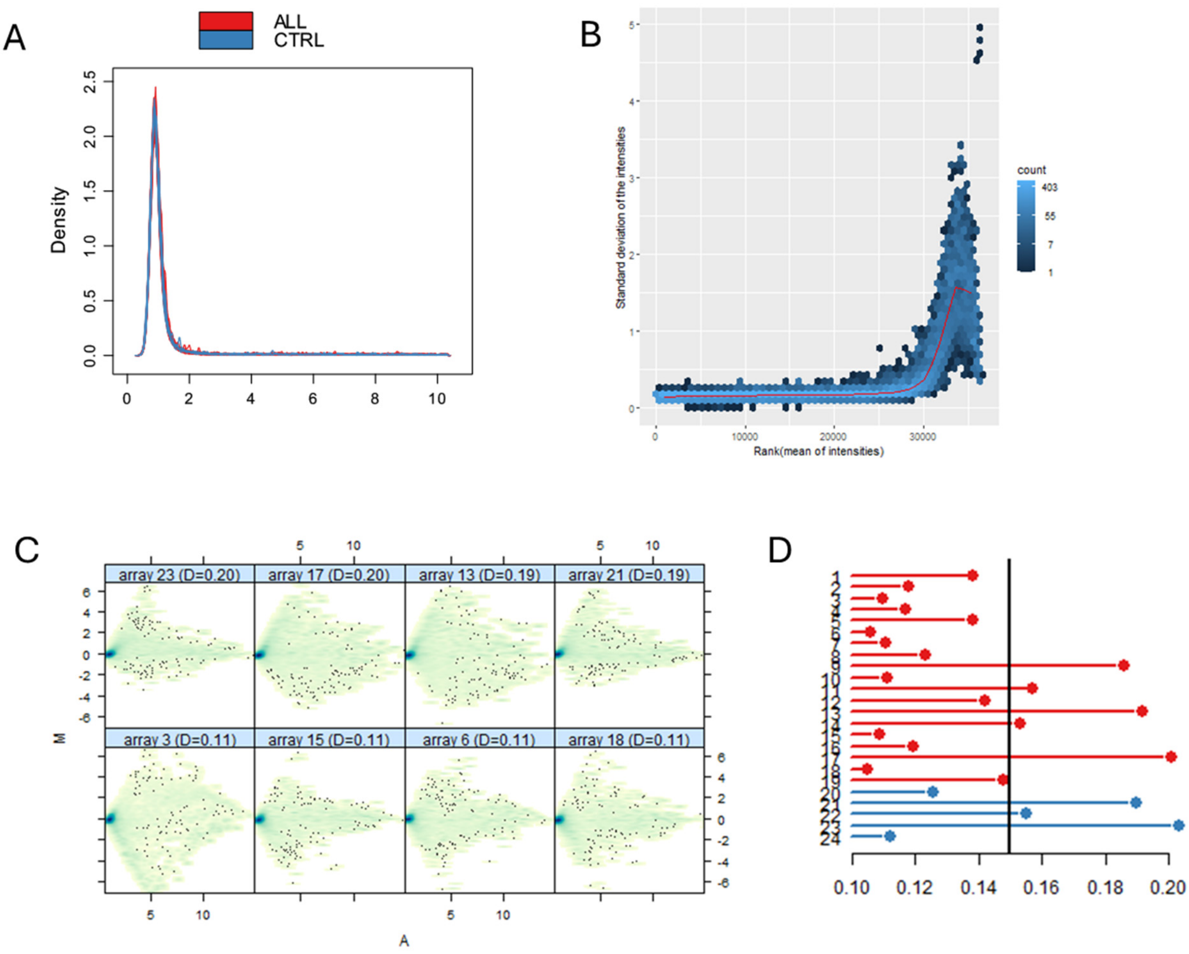

Figure S2. Quality metrics plots before exclusion (n = 24). (**A**) Density plots showing smoothed histograms for each array. The density curves largely overlap with no strong background noise, indicating no signal loss. (**B**) Standard deviation vs. mean rank. The running median is plotted in red and is a mostly flat red line, indicating good variance stabilization with no large hump at high intensities. No saturation without upward drift indicates that normalization worked properly. (**C**) MA plots: MA clouds appear centered around M = 0. There are no trending patterns (no obvious background bias or saturation). (D) Outlier Detection from MA Plots (Hoeffding's D outlier detection): Threshold = 0.15. Arrays exceeding the threshold were flagged for review by the QC algorithm.
