# Supplementary material for: Differential Expression and Target Gene Analysis of PBMC-Derived microRNAs as Prognostic Biomarkers in Acute Lymphoblastic Leukemia
Source: Int J Mol Sci. 2026 Apr 27;27(9):3868. doi: 10.3390/ijms27093868 (PMC13163416; doi:10.3390/ijms27093868)
Supplement: Supplementary file 1 [file ijms-27-03868-s001.zip › Informed Consent Blank version.pdf]

إذن خطي بالموافقة على المشاركة في البحث الطبي

Consent for the Agreement in participation in medical research

**King Abdul-Aziz University**<br>**Hospital**<br>**Psychiatry Division**	جامعة الملك عبد العزيز<br>المستشفى الجامعي
**Research Proposal:**<br><br>**Track miRNAs and T cell immunoglobulin and mucin domain-3 (Tim-3) expression in Acute Lymphocytic Leukaemia (ALL)**	**عنوان الدراسة:**<br><br>**تتبع تعبير كل من miRNAs و Tim-3 في مرض اللوكيميا الليمفاوية الحادة**
**Invitation:**<br><br>You are invited to participate in a study to investigate the expression of Tim-3 in lymphoblastic leukaemia (ALL) along with the effect of miRNAs on Tim-3 expression and its impact on proliferation and survival of ALL patients. This research is conducted by a group of physicians, academic staff, and students at KAU.<br><br>You have been chosen as a participant because you are a patient with lymphoblastic leukaemia (ALL). Please read carefully and don't hesitate if you have any questions before agreeing to participate.	**دعوه للمشاركة التطوعية:**<br><br>أنت مدعو للمشاركة في دراسة للتحقيق في التعبير عن Tim-3 في ابيضاض الدم الليمفاوي (ALL) اضافة الى تأثير miRNAs على عرض Tim-3 وتأثيره على انتشار المرض. يتم إجراء هذا البحث من قبل مجموعة من الأطباء والموظفين الأكاديميين والطلاب في جامعة الملك عبدالعزيز.<br><br>لقد تم اختيارك كمشارك لأنك مريض بسرطان الدم الليمفاوي (ALL). يرجى القراءة بعناية ولا تتردد إذا كان لديك أي أسئلة قبل الموافقة على المشاركة.
**Aims:**<br><br>To evaluate the expression of Tim-3 in ALL patients and identify some of the underlying regulatory miRNAs that resulted in cancer escaping immune destruction.	**الهدف من البحث:**<br><br>لتقييم التعبير عن **Tim-3** في جميع المرضى وتحديد بعض **miRNAs** التنظيمية الأساسية والذي يؤدي إلى هروب السرطان من التدمير المناعي.

**Study Procedures:**<br><br>If you decide to Participate in this study, we are going to explain in detail the study procedure and follow up.<br><br>Furthermore, we will contact you by phone or during your clinic visit.	**الإجراءات:**<br><br>في حال قبولك للمشاركة في هذا البحث سوف نقوم بشرح اجراءات الدراسة والمتابعة.<br><br>سوف يتواصل معك أحد المشاركين بالبحث اما بالهاتف الجوال او اثناء زيارتكم للعيادات الخارجية.
**Benefit**:<br><br>There's no direct benefit to the participant but participating will help in clarifying the pattern of presentation disease.<br><br>Participation in research does not in any way affect the medical care provided to you.	**الفائدة:**<br><br>لا توجد فائدة عائدة للمشارك بشكل مباشر، لكن المشاركة في هذا البحث تسهم في فهم المناعة ضد السرطان بشكل أفضل.<br><br>المشاركة في البحث لا تأثر بأي شكل من الأشكال على الرعاية الطبية المقدمة لكم.
**Confidentially:**<br><br>Confidentially will be maintained by de-identifying all of your personal data and we will give a code (study I.D number) for each subject enrolled in the study.	**الخصوصية:**<br><br>سيتم المحافظة على السرية التامة عن طريق إزالة جميع المعلومات الشخصية للمشاركة بالبحث وسيتم الرمز لكل مشارك برقم خاص.
**Withdrawal from the study:**<br><br>Your participation in this study is entirely voluntary and you may refuse to participate or withdraw from the study at any time without jeopardy to your privileges and access to health care.	**الانسحاب من الدراسة:**<br><br>إن مشاركتك في هذه الدراسة طوعية ولك الحق الكامل في رفض المشاركة أو الانسحاب من الدراسة في أي وقت من دون أن يؤثر هذا على الرعاية الطبية المقدمة لك.
Do you consent to participate in this study?<br><br>Yes ( ) No( )<br><br>For parents : I consent( ) do not consent ( ) to my child's participation in this study.<br>Can we use what is left from your sample for future study? Yes ( ) No( )	هل توافق على المشاركة في هذه الدراسة؟<br><br>نعم ( ) لا ( )<br><br>للوالدين: هل توافق نعم ( ) ترفض ( ) مشاركة طفلك في هذه الدراسة.<br><br>هل توافق على استخدام بياناتكم لدراسة مستقبلية والنشر بدون ذكر أسمك؟<br><br>نعم ( ) لا ( )

**Your signature indicates that you have received a copy of this consent.**	**توقيعك يثبت استلامك نسخة من الموافقة**
**Name of Patient**:<br><br>**Date:**<br><br>**Signature of the patient or his guardian:**<br><br>**Name of Researcher:**<br><br>**Date:**<br><br>**Signature:**	**الاسم الكامل للمشارك:**<br><br>**التاريخ:**<br><br>**توقيع المشترك أو ولي الأمر:**<br><br>**الاسم الكامل للباحث الرئيس أو أحد فريق البحث:**<br><br>**التاريخ:**<br><br>**توقيع الباحث:**
